# Correction to: A systematic review of the effectiveness of community interventions to improve parent health literacy

**DOI:** 10.1093/eurpub/ckad113

**Published:** 2023-07-27

This is a correction to: S Belfrage, M Husted, S Fraser, S Patel, J Faulkner, A systematic review of the effectiveness of community interventions to improve parent health literacy, *European Journal of Public Health*, Volume 32, Issue Supplement3, October 2022, ckac130.143, https://doi.org/10.1093/eurpub/ckac130.143

In the originally published version of this manuscript, the first author’s name was omitted, an incorrect name marked for corresponding author and the correct email contact was included under an incorrect name also. The author list should read: “S Belfrage*, M Husted, S Fraser, S Patel, J Faulkner” instead of: “M Husted*, S Fraser, S Patel, J Faulkner”. The corresponding author details have been emended to be with the correct name.

These errors have been corrected in the article.

